# Assessment of Patient Safety in a Low-Resource Health Care System: Proposal for a Multimethod Study

**DOI:** 10.2196/50532

**Published:** 2024-03-27

**Authors:** Ghazal Haque, Fozia Asif, Fasih Ali Ahmed, Farwa Ayub, Sabih ul Hassan Syed, Nousheen Akber Pradhan, Malika Hameed, Muhammad Muneeb Ullah Siddiqui, Shafaq Mahmood, Tahani Zaidi, Sameen Siddiqi, Asad Latif

**Affiliations:** 1 Center for Patient Safety Aga Khan University Karachi Pakistan; 2 Department of Community Health Sciences Aga Khan University Karachi Pakistan; 3 Department of Anesthesiology Aga Khan University Medical College Karachi Pakistan

**Keywords:** patient safety, health systems, quality assessment, safety culture, assessment, healthcare delivery, health system, hospital, low-middle-income countries, research methodology

## Abstract

**Background:**

The high prevalence of adverse events (AEs) globally in health care delivery has led to the establishment of many guidelines to enhance patient safety. However, patient safety is a relatively nascent concept in low- and middle-income countries (LMICs) where health systems are already overburdened and underresourced. This is why it is imperative to study the nuances of patient safety from a local perspective to advocate for the judicious use of scarce public health resources.

**Objective:**

This study aims to assess the status of patient safety in a health care system within a low-resource setting, using a multipronged, multimethod approach of standardized methodologies adapted to the local setting.

**Methods:**

We propose purposive sampling to include a representative mix of public and private, rural and urban, and tertiary and secondary care hospitals, preferably those ascribed to the same hospital quality standards. Six different approaches will be considered at these hospitals including (1) focus group discussions on the status quo of patient safety, (2) Hospital Survey on Patient Safety Culture, (3) Hospital Consumer Assessment of Healthcare Providers and Systems, (4) estimation of incidence of AEs identified by patients, (5) estimation of incidence of AEs via medical record review, and (6) assessment against the World Health Organization’s Patient Safety Friendly Hospital Framework via thorough reviews of existing hospital protocols and in-person surveys of the facility.

**Results:**

The abovementioned studies collectively are expected to yield significant quantifiable information on patient safety conditions in a wide range of hospitals operating within LMICs.

**Conclusions:**

A multidimensional approach is imperative to holistically assess the patient safety situation, especially in LMICs. Our low-budget, non–resource-intensive research proposal can serve as a benchmark to conduct similar studies in other health care settings within LMICs.

**International Registered Report Identifier (IRRID):**

PRR1-10.2196/50532

## Introduction

### Background

Adverse events (AEs) are instances of injury or harm to patients as a result of medical care and not their underlying medical condition [1]. They are ubiquitous, with some estimates saying that up to 1 in 10 hospitalizations involve medical errors [2]. Underreported and often overlooked, they take a heavy toll on health systems globally. In the United States alone, an estimated 250,000 deaths are attributed to AEs annually, making them the third leading cause of mortality in the country [3]. Moreover, anywhere from 25% to 80% of these errors—some of which lead to a loss of life or permanent disability—are entirely preventable [4,5]. Due to a myriad of factors including but not limited to, understaffing, resource availability and use, and lack of health literacy, health systems within low- and middle-income countries (LMICs) are estimated to experience far more AEs, with an incidence rate ranging between 2.5% and 18.4% [5].

Given these facts, it is understandable why the domain of patient safety and harm reduction gained traction around the world. Of note, the publication of the Institute of Medicine’s report “To Err is Human” in 2000 served as a benchmark in establishing the relevance of this aspect of health care delivery [6]. Since then, it has become increasingly apparent that it is a complex issue requiring a multidimensional approach. This school of thought is also linked to the relatively recent emergence of the “systems thinking” concept. This concept emphasizes recognizing the importance of a systems approach in studying the causes of patient harm and advocates for designing an error-proof system. Such an environment is aimed at preventing human errors and focuses on mitigation rather than the elimination of human factors in health care provision [7]. Additionally, an open and transparent environment where a culture of patient safety is prevalent is also imperative in fostering safe health care systems, which ultimately reduce the chances of errors and AEs. This safety culture can be nurtured by upholding safety beliefs, values, and attitudes among the majority of the workforce [8].

Moreover, viewing patient safety through a systems lens is a low-resource exercise since it mainly requires a shift in cultural and systemic perspectives. By considering patient safety problems as a product of the interaction between human and system factors, clinicians can evaluate the factors contributing to patient safety issues without the need for expensive or time-consuming resources [7]. This is particularly important for LMICs where large groups of the population are catered to using precious scarce resources and more often than not, have fewer human resources available per capita. The low cost of these measures makes the establishment of a patient safety culture a near-ideal step in achieving the provision of safe health care delivery and maximizing the quality and impact of health care services [9]. The cost-effectiveness of patient safety culture and a systems approach is reflected in the idea that prevention of AEs is much less resource-intensive than treating the complications that arise from them and which impose a heavy burden on already strained health systems [1,10].

### Knowledge Gap

Though sparse, the existing literature from LMICs suggests that there are considerable knowledge gaps in the patient safety domain despite a general awareness of its importance [11,12]. However, global awareness of patient safety has also resulted in a shift of focus toward improvement in the quality of care in LMICs [13,14]. The Global Patient Safety Collaborative is one such initiative established by the joint efforts of the World Health Organization (WHO) and the governments of the United Kingdom and Northern Ireland to scale up global efforts to prioritize patient safety and improve the safety of health systems at a country level [15].

Similarly, the WHO’s Global Patient Safety Action Plan for 2021-2030 [16] outlines some key points in eliminating preventable harm in health care through their defined goals of completely eliminating avoidable harm and ensuring the delivery of safe clinical processes, building reliable health systems to protect patients’ rights to safe and quality care, empowering both health care providers and patients by engaging in productive dialogue to influence patient safety policies, and ensuring effective information and knowledge sharing among health systems and partners to promote multidisciplinary involvement in patient safety. Therefore, to achieve a holistic understanding of the status of patient safety within a health care system, it is necessary to approach the problem simultaneously from multiple perspectives such as the health care provider’s view of patient safety, the health care consumer’s view of patient safety and AEs, the estimated incidence of AEs happening within a health care setting, and the infrastructure available to cater to these problems. To our knowledge, a unified framework that addresses all such facets of patient safety has not been used at scale, hence our proposal is unique in its approach.

As mentioned above, to establish a proper culture of safety, it is imperative to first assess the existing culture of safety within health systems [17]. The most commonly used tool for this assessment is the United States Agency for Healthcare Research and Quality’s (AHRQ) Hospital Survey on Patient Safety Culture (HSOPSC), which has been implemented in many countries [18-21], translated into several different languages, and can be adapted to fit the local context of most countries [22]. Similarly, the AHRQ’s Hospital Consumer Assessment of Healthcare Providers and Systems (HCAHPS) questionnaire is used globally to assess the patient perspective on health care provision and service delivery. Standards such as the Joint Commission International Accreditation and the WHO’s Patient Safety Friendly Hospital Framework (PSFHF) serve as benchmarks against which health care settings can be measured on their quality indicators.

LMICs can greatly benefit from initiatives like the Global Patient Safety Collaborative and PSFHF and from using tools such as the HSOPSC and HCAHPS, to perform comprehensive risk assessments of their hospitals, deliver patient safety education and training, establish a culture of safety, and expand the capacity for patient safety within their hospitals [23]. The generalizability of these standardized, validated tools makes them easily adaptable to the local context and can help evaluate patient safety standards across varying health systems. However, there is a considerable dearth of research in this domain in LMICs and to our knowledge, a comprehensive study on patient safety has not been undertaken so far in Pakistan.

With this proposal, our goals are to evaluate, develop, and implement evidence-based patient safety assessment policies and recommend patient safety strategies and plans that can be replicated across low-resource settings. In order to achieve these goals we aim to perform a comprehensive assessment of the patient safety problem at a sample of hospitals in each province in the country. Additionally, we seek to analyze the results of these assessments to establish the status of patient safety problems in local hospitals, in order to recommend practical steps and policy guidelines for improvement.

## Methods

### Patient Safety Assessment Framework

**Figure 1:**
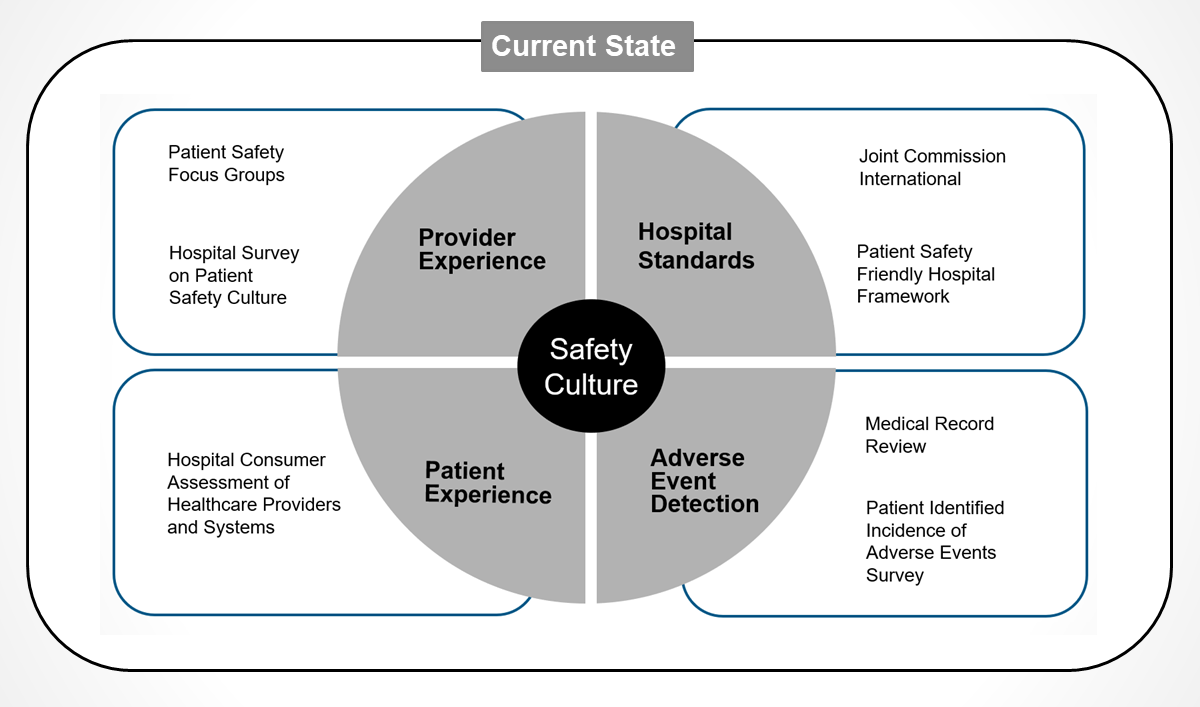
Framework for patient safety assessment.

To better comprehend the range of patient safety problems and their contributing factors at a variety of hospitals across the country, we propose a multifaceted, multimethod approach with the goal of developing a holistic understanding of patient safety issues within the local health care system. For this purpose, we have devised a conceptual framework for the holistic assessment of the patient safety status across variable health settings. Our framework for the assessment of patient safety (Figure 1) in a low-resource setting approaches the problem from a systems thinking lens. We aim to evaluate each hospital setting on its safety culture, the incidence of AEs, and existing infrastructural standards against a minimum, preset standard. After the study sites are identified and all necessary approvals are obtained, multiple methodologies will be used to analyze the patient safety situation at these sites within 3 interconnected domains: patient safety culture, AE detection, and patient safety infrastructure. We shall use 6 different approaches that can be variably used in either a multicenter study design or in individual health care settings.

### Study Site Selection

In our proposal, a cross-section of public and private hospitals providing varying levels of care to urban or rural populations will be considered for these assessments. Since there is not a single electronic health record network or a unique quality standard implemented across all hospitals in Pakistan, our aim would be to include hospitals participating in the World Health Organization’s Patient Safety Friendly Hospital Initiative. The Patient Safety Friendly Hospital Initiative involves a basic, easily implemented framework for hospital quality standards developed by the WHO Regional Office for the Eastern Mediterranean and has been successfully piloted in 7 countries including Pakistan [24]. Attempts will be made to include 1 secondary or tertiary hospital from each province to accommodate for regional variations in a decentralized health system. Following this, key personnel will be identified from the selected centers and stakeholder meetings will be conducted to get the appropriate permissions from all relevant parties on board for subsequent hospital assessments.

### Ethical Considerations

Prior to data collection, separate approvals will be obtained from the institutional research review board or an equivalent body of each participating institute. An overarching approval will also be obtained from the institution conducting the research. For study components requiring individual responses, an informed consent form in either English or a certified Urdu translation will be obtained per the respondent’s preference. Each respondent will also be offered a blank copy of the consent form.

For study components requiring medical record access, a review of the participating health center’s policy on data governance and sharing will be conducted before any medical records are requested for the study. Consent will be obtained from all relevant institutional authorities prior to medical record reviews. All identifying information will be coded to ensure strict patient confidentiality and anonymity. The collected data will be stored on secure servers with limited access provided only to authorized research personnel. All information generated will be used exclusively for research purposes in accordance with local regulations.

### Safety Culture

#### Focus Group Discussions

To assess the existing infrastructure and culture of patient safety and quality improvement at the study sites, focus groups will be conducted with teams comprising each hospital’s leadership, unit-level management, and frontline health care workers. Administrative staff, physicians, nurses, and technicians will be targeted for responses in representative proportions. During these discussions, in-depth interviews will be conducted to try to capture informal methods of health care delivery that might be in practice at each institute, to get an idea of the local understanding of patient safety, and to identify the problems associated with it. A qualitative analysis will be performed following these interviews to identify the relevant codes and themes pertaining to the patient safety situation in these hospitals. Textbox 1 shows a sample of the prompts that will be used for these interviews.

**Interview prompts for focus group discussions among health care providers on the status of patient safety.**

**Prompt:**

- What is your understanding of quality and patient safety?
- What processes/activities/mechanism currently exist at your hospital for quality improvement and patient safety?
- Please share the last unexpected/adverse event that you have encountered/observed at your hospital
- What activities (if any) are planned for initiating/strengthening the existing quality and patient safety culture at the hospital?
- What are your suggestions to improve/strengthen patient safety at your hospital?

#### Evaluation of Existing Patient Safety Culture

To evaluate the existing safety culture in the participating hospitals, a survey will be conducted using the Agency for Healthcare Research and Quality’s HSOPSC [25]. HSOPSC is a standardized, validated tool consisting of 42 items that assess patient safety culture across 12 basic dimensions (Textbox 2). For this survey, a minimum of 2 available personnel belonging to five categories—doctors, nurses, technicians, hospital management, and hospital aides—will be included via quota sampling from the participating hospitals, with a minimum of 10 personnel per hospital. Responses to most items in this survey will be based on a Likert scale, some of which will be dichotomized during analysis to be measured against the predefined composites listed in Textbox 2.

**Hospital Survey on Patient Safety Culture (HSOPSC) survey items and composite measures.**

**Item**

- Teamwork
- Staffing and work pace
- Organizational learning—continuous improvement
- Response to error
- Supervisor, manager, or clinical leader support for patient safety
- Communication about error
- Communication openness
- Reporting patient safety events
- Hospital management support for patient safety
- Hands-off and information exchange
- Number of events reported
- Patient safety rating

Note: Survey items included in the HSOPSC are grouped by safety culture composite measures [25].

#### Inpatient Hospital Experience Survey

To measure the quality of the inpatient hospital experience from the patients’ perspective, a modified version of the AHRQ’s Hospital Consumer Assessment of Healthcare Providers and Systems questionnaire will be used to conduct an interview-based survey. The HCAHPS is a widely used standardized survey used to measure patient satisfaction with in-hospital care [26]. The standard HCAHPS survey contains 29 questions split into 7 discrete categories, which measure the patient’s experience during their hospital stay in specific areas of inpatient health care delivery. As a standard practice, it should be administered randomly to adult patients between 48 hours and 6 weeks after discharge from the hospital and a minimum of 300 patients should be surveyed per hospital, from 1 calendar year.

Our modified HCAHPS questionnaire adds one more category with additional questions on pain management, adapted from the Qatar Ministry of Public Health’s Patient Experience Survey for Hospitals (Textbox 3). Using quota sampling for this survey, we aim to target a minimum of 20 patients being discharged from each participating hospital. Most responses in the HCAHPS are measured with a 4-point Likert scale, which during analysis, will be dichotomized into binary values to elicit the highest positive responses to the items—the so-called “top box percentages.”

**Hospital Consumer Assessment of Healthcare Providers and Systems (HCAHPS) survey question categories.**

**Item:**

- Nurse communication
- Doctor communication
- Hospital environment
- In-hospital care experience
- Pain management
- Discharge or transfer information
- Overall hospital rating
- Understanding your care transition upon discharge

Note: Survey categories are based on a standard HCAHPS survey form modified to include questions on pain management [26].

### Adverse Event Detection

#### Patient–Reported Incidence of AE

To understand the patients’ perspective of AEs during health care delivery, we propose a questionnaire-based survey to assess patient safety–related issues including patients’ understanding and experiences of AEs, preventable harm, and local reporting. This survey is adapted from a tool implemented by Southwick et al [27] to suit the local health care setting. The original study design surveyed almost 700 patients in nearly a 4-year period through a web-based form.

To account for low digital literacy within LMICs we propose using quota sampling to screen patients within the outpatient departments at the study sites to survey those who have availed health care services. Participants will be requested to recall potential AEs experienced during health care delivery using the standardized questionnaire, and the responses will be recorded by a member of the investigating team. The results will be analyzed descriptively to determine the nature and severity of AEs, and their effects as perceived by patients who have received medical care. At least 50 respondents will be interviewed at each study site.

#### Medical Record–Based Incidence of AEs

To calculate the incidence of AEs during hospitalization, a review of medical records will be performed at each hospital. The sample size for this study will be calculated based on the annual inpatient volume at each hospital with a 5% significance, a precision of 3%, and an estimated 10% dropout rate due to unavailability or poor quality of medical records. A range of prevalence of 10%-18% will be assumed as representative of the population. A team of investigators will also perform a data quality check of the existing medical records against a standardized checklist prior to the review [28].

Following this, a retrospective chart review of the medical records will be conducted in a 2-step process. In the first step, all charts will be screened using a standardized AE screening form, “Review Form 1” (RF1) to identify any potential AEs. All AEs identified using RF1 will then be evaluated using “Review Form 2” (RF2) to establish the nature, causality, and the factors contributing to these AEs. Both review forms have been adapted from the WHO patient safety research tools for data-poor hospitals and contain extensive screening and investigative questions to assess and analyze “harmful incidents” or “adverse events” [29]. The incidence of AEs will be calculated using the following formula:



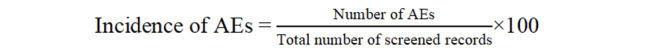



Additionally, a descriptive analysis will be performed on the types of AEs, their potential causes, and the likely systemic and human factors contributing to the event.

### Patient Safety Infrastructure Assessment Against the WHO’s Patient Safety Friendly Hospital Framework

The PSFHF is based primarily on 5 domains and 22 standards [30], which together comprise 134 criteria that are prioritized into critical, core, and developmental categories (Table 1).

The 25 critical criteria are the basic minimum requirements that hospitals are encouraged to achieve in terms of quality improvement. To assess each hospital’s standing against the PSFHF criteria, members of the investigating team will conduct in-person surveys of the facilities to observe the implementation of standards. They will also review standard operating procedures and existing hospital protocol documents, and check for the existence of an AE reporting system. Interviews with staff members and patients will also be conducted. All these observations will be recorded against the existing standard criteria set within the PSFHF and after a comprehensive review, a list of recommendations will be provided by the investigators in light of their findings.

**Table 1:** Attributes of the Patient Safety Friendly Hospital Network (PSFHF).

Domain	Standards, n	Critical criteria, n	Core criteria, n	Developmental criteria, n	Total criteria, n
Leadership and management	6	7	26	3	36
Patient and public involvement	7	2	22	7	31
Safe evidence–based clinical practice	4	14	24	2	40
Safe environment	2	1	20	1	22
Lifelong learning	3	1	2	2	5
Total	22	25	94	15	134

### Project Execution

**Figure 2:**
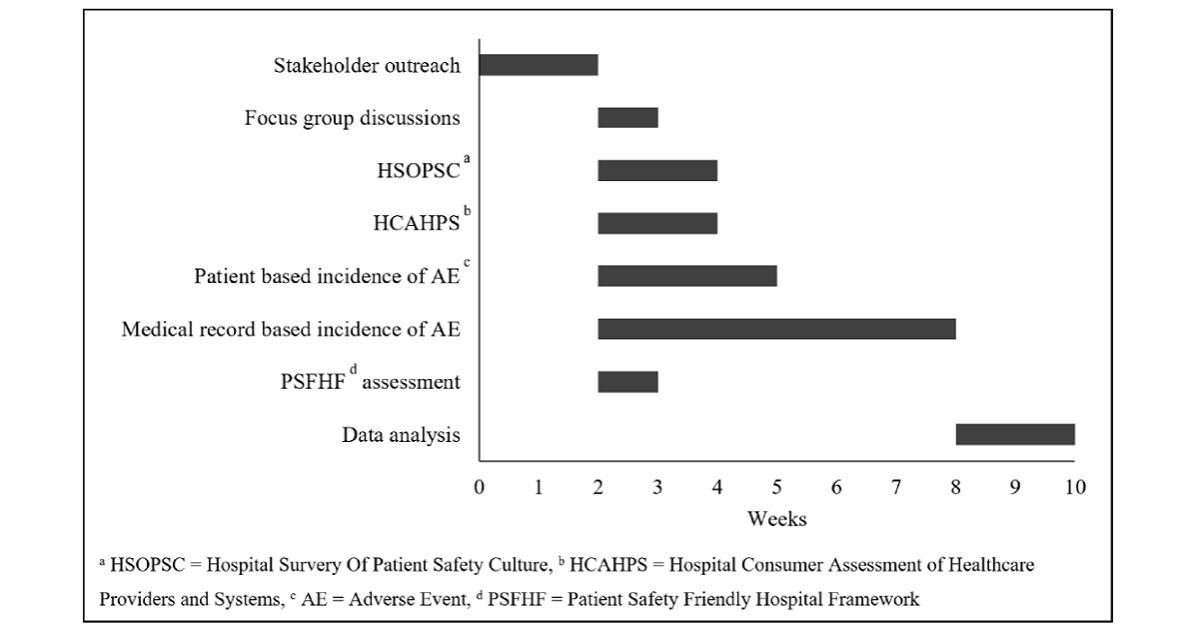
Proposed project timeline of events for a single study site.

In order to conduct all the above study components in an organized and timely manner, the investigating team will comprise health care professionals recruited from the core team and regional partners, who will be trained in conducting each survey. All survey instruments will be developed and recorded in English, whereas the written consent forms for participant recruitment will be translated into the local language as well. The interviewers will be fluent in both languages. The proposed timeline of events for conducting each component of the assessment is given in Figure 2.

It should be noted that as part of a theoretical framework, this proposed timeline includes a timeframe for the completion of each part of this multimodal approach, carried out simultaneously by multiple teams at each site. However, the authors understand that various levels of care within various health care systems require different facets of evaluation. Thus, it is recommended that the approach within this framework be modified according to the local organizational and operational structures and the given limitations of the health care setting being assessed. Subsequently, the project timeline can vary depending on the number of assessments being conducted.

## Results

A thorough quantitative and qualitative analysis will be conducted which will be aimed for publication in the form of a technical report.

## Discussion

### Overview

Patient safety culture is an essential component of health care provision and can significantly impact patient outcomes. The beliefs, attitudes, and behaviors of health care providers can significantly affect their understanding of and commitment to ensuring patient safety [10]. Research has consistently shown that a positive patient safety culture leads to improved patient outcomes, increased staff satisfaction, and reduced health care costs [31]. Conversely, a lack of an open, transparent culture focused on patient safety can result in medical errors, AEs, and poor patient outcomes [17]. LMICs in particular fall behind in prioritizing patient safety due to a lack of practical policies and procedures, inadequate education and training, and lacking a culture of transparency and open communication to ensure the best possible patient outcomes [5]. These are largely attributed to the chronically depleted health resources in the region, however, establishing a culture of safety does not necessarily require high-cost interventions. Additionally, in order to establish a sustainable culture of safety, it is essential to first identify the gaps that lie within this system.

Studying the incidence and prevalence of AEs within a health system is an effective way to gauge the status of patient safety. Historically, this has been achieved using incident reporting systems and retrospective chart reviews to record error prevalence [32,33], health consumer and provider surveys [19,23,34], and by studying medical litigation cases [35]. There is a considerable amount of literature from high-income countries that highlights the incidence and impact of AEs in health care [2,27,36], however, the same is not true for LMICs [5]. Moreover, patient safety problems are multifactorial and therefore require a diverse yet intertwined approach to obtain a comprehensive understanding of the problems involved, such as system-based issues, which tend to be locally unique at the unit, hospital, or even regional level. For example, an inpatient hospital setup might have patient safety concerns due to culture issues, while the emergency room at the same hospital might have patient safety concerns due to inadequate resources for their volumes. Similarly, the same type of work area in variably resourced settings, such as the intensive care unit in a public versus a private hospital might have vastly different reasons for gaps in patient safety. Therefore, a comprehensive risk assessment of factors contributing to patient safety concerns is the first step in identifying these issues and subsequently addressing them.

Therefore, our proposed multimodal methodology for assessing patient safety issues in the Pakistani health care system is a pragmatic approach to the problem. With the participation of local and federal stakeholders, the application of our research proposal is easily achievable at a very low cost to the health system. Estimating the incidence of AEs from retrospective chart reviews and patient interviews, assessing the presence of a safety culture within hospitals by interviewing health care providers, learning from patient experiences within the hospital through surveys, and examining hospital compliance to quality improvement measures are all technically sound methods to ascertain the dynamics and challenges within patient safety culture.

### Challenges and Solutions

However, even with a less resource-intensive approach such as ours, the local health system still poses many challenges. Implementation of patient safety protocols for quality health care provision in LMICs is limited, mainly due to the unavailability of resources and proper infrastructure. A significant number of health care facilities in Pakistan lack essential equipment, life-saving medications, and adequately trained staff to provide safe and effective care to patients [37]. Additionally, given the diversity in the local population in terms of language and regional lifestyle differences, health care providers can encounter cultural and linguistic barriers that hinder their ability to communicate effectively with patients, which can subsequently compromise patient safety.

Furthermore, the lack of access to continuing medical and nursing education and training opportunities results in poor understanding and implementation of patient safety protocols among health care providers. Additionally, a decentralized health care system paired with political instability and poor governance frequently results in inadequate funding [37] and a dearth of regulations for implementing patient safety protocols effectively. Consequently, health care providers in Pakistan continually navigate these multifaceted challenges while striving for the provision of safe, effective, and quality care for their patients [38].

We anticipate all these issues to surface during our research project as well. To begin with, a significant challenge in collaborative risk assessments in a decentralized health system is to have all the various stakeholders agree on a singular model. Additionally, the lack of a common understanding of the concept of patient safety risk assessment results in discrepancies in the identification and management of risks by the health care providers at different institutions. Moreover, limited channels of communication among the local health care systems also mean that effective collaboration and information sharing among health care providers is almost nonexistent.

Another significant challenge in our research will likely be the absence or inadequacy of medical records within the local hospitals. Poor medical records result in incomplete or incorrect information about medical histories, leading to inaccurate diagnoses, inappropriate treatment, and consequently, AEs. Predictably, poor medical records might limit our ability to track patient progress and treatment outcomes, which can hinder the identification and management of AEs in a retrospective analysis of medical errors and near-misses.

In order to mitigate these problems head-on, we shall aim to diligently communicate with all federal and provincial stakeholders, listen to their concerns, and with the help of our subject matter experts, express our research ideas, intentions, and expected outcomes as transparently as possible. Additionally, the investigating team will include regional partners who can provide valuable cultural and linguistic context to the data collection process. To streamline the project focus, we shall invite all the major stakeholders to a brainstorming session where their perceptions and practices regarding patient safety will be discussed and incorporated within the data analysis. To combat the problem of poor quality of medical records, all reviewed charts will undergo a quality assessment [28] before they are included in the data set and a wider margin for dropouts will be adjusted in the sample size calculation if needed. Finally, given the law-and-order situation in the country, all efforts will be made to ensure the physical security of the investigating team and the project data.

Our patient- and provider-centered approach to patient safety assessment incorporates the Global Patient Safety Action Plan goals [16] of empowering patients, encouraging health care workers to participate in fostering a culture of safety, sharing valuable information across health systems, promoting transparency in incidence event reporting and making health care delivery safer to produce better outcomes. Building on this knowledge of the patient safety status in Pakistan, we hope to inform and inspire policy making and strive to align local patient safety standards with global recommendations.

### Strengths and Limitations

Our research proposal has many strengths. Given the proposed diversity of health care facilities in our study sample, and their varying capacities for health care provision, no single assessment tool can provide an exhaustive description of the patient safety situation in these hospitals. Hence, our diverse multimethod approach is not only unique within patient safety research in LMICs, but it will also provide a comprehensive assessment of the situation. The inclusion of patient safety and quality experts within the research group is also a unique feature for a survey of this magnitude. The relatively low cost of our proposed methodology and short execution time will encourage stakeholder interest in the project. Moreover, our final analysis is expected to provide a first-of-its-kind perspective on the patient safety situation, particularly within the public health care system.

Some of the limitations of our study include the significant disparity in the perception of health economics and literacy in the study population. This can result in a wide shift in the perception of quality indicators in health care between high and low-income countries. This requires adjusting the existing global standards in quality health care delivery against the local perception of it. Moreover, the reliance on retrospective chart reviews limits information availability, while patient interviews introduce the possibility of respondent recall bias in assessing the incidence of AEs. Additionally, some geographical areas of interest for our study might not be logistically or politically feasible for inclusion. There might also be concerns among the participating hospitals regarding data sharing with other institutions. However, all efforts will be made to ensure compliance with confidentiality standards.

### Conclusions

Our multidimensional, multimethod research proposal to assess and analyze the patient safety situation on a large scale within the Pakistani health care system is a unique approach to broaching the domain of patient safety in the country. We are confident that our methodology will produce good quality data so that we can use our study results in writing situation analyses, offering policy recommendations, and hopefully, instigating some real change in prioritizing and implementing a safety culture in Pakistani hospitals. Moreover, our research proposal can be easily implemented in other LMICs with a few minor adjustments in the local context.

## Abbreviations

AE: adverse event
AHRQ: Agency for Healthcare Research and Quality
HCAHPS: Hospital Consumer Assessment of Healthcare Providers and Systems
HSOPSC: Hospital Survey on Patient Safety Culture
LMIC: low and middle–income country
PSFHF: Patient Safety Friendly Hospital Framework
RF1: review form 1
RF2: review form 2
WHO: World Health Organization

## Data Availability

Data sharing is not applicable to this article as no data sets were generated or analyzed during this study.

## References

[1] Rodziewicz TL, Houseman B, Hipskind JE (2023). Medical error reduction and prevention. StatPearls.

[2] de Vries EN, Ramrattan MA, Smorenburg SM, Gouma DJ, Boermeester MA (2008). The incidence and nature of in-hospital adverse events: a systematic review. Qual Saf Health Care.

[3] Makary MA, Daniel M (2016). Medical error-the third leading cause of death in the US. BMJ.

[4] Schwendimann R, Blatter C, Dhaini S, Simon M, Ausserhofer D (2018). The occurrence, types, consequences and preventability of in-hospital adverse events - a scoping review. BMC Health Serv Res.

[5] Wilson RM, Michel P, Olsen S, Gibberd RW, Vincent C, El-Assady R, Rasslan O, Qsous S, Macharia WM, Sahel A, Whittaker S, Abdo-Ali M, Letaief M, Ahmed NA, Abdellatif A, Larizgoitia I (2012). Patient safety in developing countries: retrospective estimation of scale and nature of harm to patients in hospital. BMJ.

[6] Kohn LT, Corrigan JM, Donaldson MS, Institute of Medicine Committee on Quality of Health Care in America (2000). To Err is Human: Building a Safer Health System.

[7] Carayon P, Wooldridge A, Hoonakker P, Hundt AS, Kelly MM (2020). SEIPS 3.0: Human-centered design of the patient journey for patient safety. Appl Ergon.

[8] Guldenmund FW (2010). (Mis)understanding safety culture and its relationship to safety management. Risk Anal.

[9] Kaur G, Prinja S, Lakshmi PVM, Downey L, Sharma D, Teerawattananon Y (2019). Criteria used for priority-setting for public health resource allocation in low- and middle-income countries: a systematic review. Int J Technol Assess Health Care.

[10] Bates DW, Singh H (2018). Two decades since to err is human: an assessment of progress and emerging priorities in patient safety. Health Aff (Millwood).

[11] Nabilou B, Feizi A, Seyedin H (2015). Patient safety in medical education: students' perceptions, knowledge and attitudes. PLoS One.

[12] Shah N, Jawaid M, Shah N, Ali SM (2015). Patient safety: perceptions of medical students of Dow Medical College, Karachi. J Pak Med Assoc.

[13] (2017). Patient Safety: Making Health Care Safer. World Health Organization.

[14] The Lancet (2012). The struggle for universal health coverage. Lancet.

[15] (2019). Global patient safety collaborative. World Health Organization.

[16] (2021). Global Patient Safety Action Plan 2021-2030: Towards Eliminating Avoidable Harm in Health Care. World Health Organization.

[17] Hellings J, Schrooten W, Klazinga N, Vleugels A (2007). Challenging patient safety culture: survey results. Int J Health Care Qual Assur.

[18] El-Jardali F, Jaafar M, Dimassi H, Jamal D, Hamdan R (2010). The current state of patient safety culture in Lebanese hospitals: a study at baseline. Int J Qual Health Care.

[19] Chen IC, Li HH (2010). Measuring patient safety culture in Taiwan using the Hospital Survey on Patient Safety Culture (HSOPSC). BMC Health Serv Res.

[20] Waterson P, Griffiths P, Stride C, Murphy J, Hignett S (2010). Psychometric properties of the hospital survey on patient safety culture: findings from the UK. Qual Saf Health Care.

[21] Bodur S, Filiz E (2010). Validity and reliability of Turkish version of "Hospital Survey on Patient Safety Culture" and perception of patient safety in public hospitals in Turkey. BMC Health Serv Res.

[22] Palmieri PA, Leyva-Moral JM, Camacho-Rodriguez DE, Granel-Gimenez N, Ford EW, Mathieson KM, Leafman JS (2020). Hospital survey on patient safety culture (HSOPSC): a multi-method approach for target-language instrument translation, adaptation, and validation to improve the equivalence of meaning for cross-cultural research. BMC Nurs.

[23] Ahmed FA, Asif F, Munir T, Halim MS, Feroze Ali Zehra, Belgaumi A, Zafar H, Latif A (2023). Measuring the patient safety culture at a tertiary care hospital in Pakistan using the Hospital Survey on Patient Safety Culture (HSOPSC). BMJ Open Qual.

[24] Siddiqi S, Elasady R, Khorshid I, Fortune T, Leotsakos A, Letaief M, Qsoos S, Aman R, Mandhari A, Sahel A, El-Tehewy M, Abdellatif A (2012). Patient safety friendly hospital initiative: from evidence to action in seven developing country hospitals. Int J Qual Health Care.

[25] Yount N, Sorra J, Famolaro T, Gray L, Rockville W (2021). Hospital Survey on Patient Safety Culture Version 2.0: User's Guide.

[26] Hospital Consumer Assessment of Healthcare Providers and Systems (HCAHPS).

[27] Southwick FS, Cranley NM, Hallisy JA (2015). A patient-initiated voluntary online survey of adverse medical events: the perspective of 696 injured patients and families. BMJ Qual Saf.

[28] (2020). Joint Commission International Accreditation Standards for Hospitals: Including Standards for Academic Medical Center Hospitals. Joint Commission International.

[29] (2010). Assessing and Tackling Patient Harm: A Methodological Guide for Data-Poor Hospitals. World Health Organization.

[30] (2020). Patient Safety Assessment Manual: Third Edition. World Health Organization Regional Office for the Eastern Mediterranean.

[31] Meterko M, Mohr DC, Young GJ (2004). Teamwork culture and patient satisfaction in hospitals. Med Care.

[32] de Feijter JM, de Grave WS, Muijtjens AM, Scherpbier AJJA, Koopmans RP (2012). A comprehensive overview of medical error in hospitals using incident-reporting systems, patient complaints and chart review of inpatient deaths. PLoS One.

[33] Zanetti ACB, Gabriel CS, Dias BM, Bernardes A, Moura AA, Gabriel AB, Lima Júnior AJ (2020). Assessment of the incidence and preventability of adverse events in hospitals: an integrative review. Rev Gaucha Enferm.

[34] Kiaei MZ, Ziaee A, Mohebbifar R, Khoshtarkib H, Ghanati E, Ahmadzadeh A, Teymoori S, Khosravizadeh O, Zieaeeha M (2016). Patient safety culture in teaching hospitals in Iran: assessment by the Hospital Survey on Patient Safety Culture (HSOPSC). Health Manag Inf Sci.

[35] Oyebode F (2013). Clinical errors and medical negligence. Med Princ Pract.

[36] Rafter N, Hickey A, Conroy RM, Condell S, O'Connor P, Vaughan D, Walsh G, Williams DJ (2017). The Irish National Adverse Events Study (INAES): the frequency and nature of adverse events in Irish hospitals-a retrospective record review study. BMJ Qual Saf.

[37] Kurji Z, Premani ZS, Mithani Y (2016). Analysis of the health care system of Pakistan: lessons learnt and way forward. J Ayub Med Coll Abbottabad.

[38] Bashir S, Nasir M, Grasic K, Moulin M, Ali S (2022). Association between efficiency and quality of care of public healthcare facilities: evidence from Pakistan. Int J Health Plann Manage.

